# Factors Influencing Blood Component Availability: The Views of Transfusion Service Leaders in Riyadh, Saudi Arabia

**DOI:** 10.7759/cureus.111422

**Published:** 2026-06-24

**Authors:** Abdullah A AlShammari

**Affiliations:** 1 Haematopathology and Transfusion Medicine, King Saud University, Riyadh, SAU

**Keywords:** blood bank, blood component availability, blood component shortage, blood service leadership, blood shortage, healthcare leadership, patient blood management, transfusion, transfusion medicine, transfusion service

## Abstract

Background: Maintaining the availability of blood components remains a major challenge for transfusion services and healthcare institutions. Blood shortages can disrupt clinical care, complicate prioritization decisions, and place substantial operational pressure on transfusion services. Limited attention has been given to the perspectives of transfusion service personnel directly involved in shortage management, particularly within tertiary healthcare settings in Riyadh, Saudi Arabia.

Methods: A cross-sectional questionnaire-based study was conducted using a structured survey designed to assess respondent and institutional characteristics, perception of blood component shortage, practices implemented during shortage, barriers affecting shortage management, and perceived strategies to improve blood component availability. A total of 94 responses were collected from transfusion service personnel working in healthcare institutions in Riyadh. Of these, 64 respondents who were directly involved in shortage management were included in the principal analytical sample. Data were analyzed using IBM SPSS Statistics for Windows, Version 26.0 (IBM Corp., Armonk, New York, United States). Descriptive statistics were used to summarize demographic, institutional, and item-level response patterns. Composite domain scores were calculated, and reliability was assessed using Cronbach's alpha. Subgroup analyses were conducted according to age, years of experience, academic qualification, and role in shortage management.

Results: Blood component shortage was perceived as a meaningful and recurrent operational challenge. The mean composite scores were 3.68±0.90 for shortage perception, 4.03±0.52 for practices during shortage, 3.69±1.06 for barriers and constraints, and 4.44±0.73 for improvement strategies. The most strongly endorsed shortage-related experiences were the need for regular coordination with clinical departments and hospital leadership and the prioritization of patients or procedures during shortage periods. The most frequently reported practices included intensification of donor recruitment, close monitoring of blood inventory, and recall of repeat donors. Major perceived barriers included limited donor availability, high workload, insufficient staffing, high transfusion demand, and limited decision-making authority. Respondents showed the strongest agreement regarding improvement strategies, particularly increasing public awareness of blood donation, implementation of patient blood management programs, and establishment of a national blood service system. Postgraduate-qualified respondents demonstrated significantly higher shortage perception and stronger recognition of selected barriers.

Conclusions: Blood component shortage in tertiary healthcare settings in Riyadh is perceived not merely as a donor supply issue but as a broader system-level challenge shaped by operational demands, workforce capacity, governance limitations, and institutional coordination requirements. These findings support the need for integrated interventions combining donor awareness, patient blood management, workforce strengthening, and system-level coordination to improve the resilience and sustainability of blood component availability.

## Introduction

The availability of blood and blood components is a fundamental requirement for the safe and effective delivery of modern healthcare. Blood transfusion plays a critical role in the management of a wide range of clinical conditions, including acute hemorrhage, major surgical procedures, hematological disorders, oncological treatments, and critical care support. Ensuring a stable and adequate blood supply is therefore a central responsibility of transfusion services and healthcare systems. However, maintaining this balance between supply and demand remains a persistent challenge worldwide [1-4]. 

Blood component shortages have been increasingly recognized as a significant issue affecting healthcare systems across different settings. These shortages may arise due to multiple factors, including fluctuations in donor availability, seasonal variations, public health emergencies, logistical limitations, and increasing clinical demand. In recent years, global events such as pandemics and disruptions in routine healthcare services have further highlighted the vulnerability of blood supply systems [1-4]. Even in well-resourced settings, maintaining consistent availability of blood components can be difficult, particularly in institutions with high transfusion demand [5-11].

Traditionally, blood shortage has been viewed primarily as a problem of insufficient donor supply. Consequently, many interventions have focused on increasing blood donation through awareness campaigns, recruitment strategies, and donor retention programs [5-9]. While these approaches remain essential, emerging evidence suggests that blood shortage is not solely a supply-side issue. Instead, it reflects a more complex interaction between donor availability, clinical utilization, operational capacity, and system-level organization [5,8,10,11].

In this context, transfusion services are increasingly required to adopt a more comprehensive approach to blood management. This includes not only maintaining adequate supply but also optimizing utilization through strategies such as patient blood management (PBM), improving inventory control, strengthening coordination between clinical departments, and enhancing institutional and national-level planning [5,8-12]. Such approaches recognize that blood is a limited and valuable resource that must be managed efficiently across the entire healthcare system [10-12].

Despite growing recognition of the complexity of blood shortage, much of the existing literature has focused on individual components of the problem, such as donor behavior, inventory management, or clinical utilization [6-12]. Comparatively fewer studies have explored the issue from the perspective of transfusion service personnel who are directly responsible for managing shortages in real-world settings [10,11]. This perspective is particularly important, as these individuals are uniquely positioned at the interface between supply, demand, and clinical decision-making.

Furthermore, there is limited published data examining these issues within the context of Saudi Arabia, and more specifically within tertiary healthcare institutions in Riyadh, where transfusion demand is often high due to the concentration of specialized services, including oncology, hematology, trauma care, and major surgical procedures [1-5]. The operational challenges faced by transfusion services in such settings may differ from those reported in other regions, highlighting the need for context-specific research [1-5,10,11].

Understanding how transfusion service leaders and personnel perceive blood shortages, how they respond operationally, what barriers they encounter, and what strategies they consider most effective is essential for developing targeted and practical interventions. Such insights can inform both institutional practices and broader healthcare policies aimed at improving blood component availability [8-12].

Therefore, the present study was conducted to investigate the factors influencing the availability of blood components from the perspective of transfusion service personnel involved in shortage management in Riyadh. Specifically, the study aims to examine the perception of blood component shortage among transfusion service personnel, the practices implemented during shortage situations, the barriers and constraints affecting effective shortage management, and the strategies perceived as most effective for improving blood component availability.

By addressing these objectives, this study seeks to provide a comprehensive understanding of blood component shortage as a multi-dimensional healthcare challenge and to contribute evidence that may support the development of more effective, system-level approaches to ensuring sustainable blood availability [8-12].

## Materials and methods

Study design and setting

This study was conducted as a cross-sectional, questionnaire-based survey designed to assess factors influencing the availability of blood components from the perspective of transfusion service personnel. The study was carried out in tertiary healthcare institutions in Riyadh, Saudi Arabia, where transfusion services play a central role in supporting high-demand clinical specialties, including hematology, oncology, surgery, and critical care.

The cross-sectional design was selected to capture a snapshot of current practices, perceptions, and challenges related to blood component shortage management within the study population.

Study population and sampling

The target population consisted of healthcare professionals involved in transfusion services and blood bank operations, particularly those with responsibilities related to decision-making, coordination, or implementation of shortage management strategies.

Eligible participants included transfusion medicine consultants, blood bank directors and supervisors, senior laboratory technologists, and physicians involved in transfusion-related decision-making.

Participants were recruited using a convenience sampling approach, targeting individuals working in transfusion services within Riyadh-based healthcare institutions. Recruitment was conducted through the direct distribution of paper-based questionnaires and electronic distribution via online survey platforms.

This approach allowed the inclusion of participants who were not physically accessible during the data collection period.

Data collection tool

Data were collected using a structured, self-administered questionnaire developed based on a literature review and expert input in transfusion medicine. The questionnaire was reviewed and refined in consultation with a senior transfusion medicine consultant to ensure content validity and relevance to clinical practice.

The questionnaire was developed based on the study objectives, relevant literature, and expert review. Internal consistency of the questionnaire domains was subsequently assessed using Cronbach's alpha, as reported in the Results section.

The final questionnaire consisted of five main sections, as shown in Table 1.

**Table 1:** Structure and content of the study questionnaire

Section	Content
Demographic and institutional characteristics	Age, gender, academic qualification, years of experience, institutional blood supply model, and professional role and level of involvement in shortage management
Perception of blood component shortage	Frequency and impact of shortages, operational consequences such as coordination and prioritization, and the role of shortage in routine practice
Practices during shortage	Operational strategies implemented during shortage situations, including supply-side and demand-side interventions
Barriers and constraints	Factors limiting effective shortage management, including workforce-, supply-, demand-, and governance-related challenges
Improvement strategies	Respondents' views on potential interventions to improve blood availability

Items in Sections 2-5 were measured using a five-point Likert scale, as shown in Table 2.

**Table 2:** Five-point Likert scale used in Sections 2-5

Score	Response option
1	Strongly disagree
2	Disagree
3	Neutral
4	Agree
5	Strongly agree

The questionnaire was designed with conditional logic such that respondents who indicated no involvement in shortage management did not proceed to the Likert-scale sections.

Data collection procedure

A total of 94 responses were collected during the study period. Data collection was conducted using a combination of paper-based questionnaires, which were later entered into an electronic database, and online survey forms, distributed directly to participants.

Because the questionnaire was distributed through combined paper-based and electronic methods across multiple tertiary transfusion service settings, the exact total number of individuals approached was not reliably captured; therefore, an overall formal response rate could not be calculated.

All responses were consolidated into a single dataset for analysis. Data entry was reviewed to ensure accuracy and consistency, particularly for responses transferred from paper-based forms.

Data analysis

Data were analyzed using IBM SPSS Statistics for Windows, Version 26.0 (IBM Corp., Armonk, New York, United States). 

Descriptive statistics were used to summarize the data. Categorical variables were presented as frequencies and percentages, whereas continuous and Likert-scale variables were summarized using means and standard deviations. Internal consistency of the questionnaire domains was assessed using Cronbach's alpha, and reliability was interpreted according to standard thresholds. Domain-level composite scores were calculated by averaging item responses within each section to represent overall perception in the domains of shortage perception, practices, barriers, and improvement strategies. Agreement analysis was performed by combining responses categorized as "agree" or "strongly agree" to calculate agreement percentages. Subgroup analyses were conducted according to age group, years of experience, academic qualification, and role in shortage management. Differences between groups were assessed using appropriate statistical tests within SPSS, with p-values of <0.05 considered statistically significant. Given the ordinal nature of Likert-scale data and the sample size, results were interpreted using a combination of statistical significance, directional trends, and practical relevance.

Inclusion criteria for analysis

Although 94 responses were collected, only respondents who were actively involved in blood component shortage management were included in the main analytical sections (Sections 2-5).

A total of 64 respondents met this criterion and were included in the principal analysis. Respondents who indicated no involvement in shortage management were included in descriptive statistics but excluded from inferential analysis to ensure that results reflect experience-based responses.

Ethical considerations

Participation in the study was voluntary, and respondents were informed about the purpose of the study prior to completing the questionnaire. No personally identifiable information was collected, and responses were analyzed in an anonymous and confidential manner.

The study was conducted in accordance with institutional research standards and received approval from the King Saud University Institutional Review Board (approval number: KSU-HE-26-0217).

## Results

Study sample and response characteristics

A total of 94 responses were collected and included in the dataset. Of these, 30 respondents (31.9%) indicated that they were not involved in blood component shortage management and therefore did not complete the Likert-scale sections of the questionnaire. These respondents were included in the descriptive analysis but excluded from the inferential analyses of shortage perception, practices, barriers, and improvement strategies.

The final analytical sample consisted of 64 respondents (68.1%) who were directly involved in shortage management. Among these, 41 respondents (64.1%) reported primary responsibility for shortage management, while 23 respondents (35.9%) participated as members of shortage management teams.

The demographic and institutional characteristics of the respondents are summarized in Table 3.

**Table 3:** Demographic and institutional characteristics of the respondents (n=94)

Characteristic	n (%)
Male	63 (67)
Female	31 (33)
Age 18-45 years	58 (61.7)
Age >45 years	36 (38.3)
Postgraduate qualification	49 (52.1)
Bachelor-level education or less	45 (47.9)
More than five years of experience	68 (72.3)
Mainly hospital-based blood supply system	61 (64.9)
Mixed (hospital and central) system	21 (22.3)
Mainly central/national blood supply system	12 (12.8)
Laboratory technologists	56 (59.6)
Transfusion medicine consultants	21 (22.3)
Other roles (physicians and trainees)	17 (18.1)

This distribution indicates a sample with substantial representation of experienced personnel and frontline transfusion service staff.

Reliability of questionnaire domains

The internal consistency of the questionnaire domains was assessed using Cronbach's alpha, as shown in Table 4. 

**Table 4:** Internal consistency of questionnaire domains

Domain	Cronbach's alpha
Shortage perception	0.805
Practices during shortage	0.585
Barriers and constraints	0.909
Improvement strategies	0.843

These findings indicate good to excellent reliability for most domains, supporting the use of composite scores for further analysis.

Domain-level analysis

Among respondents involved in shortage management (n=64), the composite mean scores and agreement levels for each domain are summarized in Table 5. 

**Table 5:** Domain-level composite scores and agreement levels among respondents involved in shortage management (n=64) Scale: items were rated on a five-point Likert scale where 1 means strongly disagree, 2 means disagree, 3 means neutral, 4 means agree, and 5 means strongly agree.

Domain	Mean±SD	Agreement (%)
Shortage perception	3.68±0.90	60.7
Practices during shortage	4.03±0.52	74
Barriers and constraints	3.69±1.06	63.8
Improvement strategies	4.44±0.73	86.7

These findings indicate moderate to high perception of shortage, high engagement in operational practices, persistent recognition of barriers, and very strong agreement on improvement strategies.

Perception of blood component shortage

Respondents reported that blood shortage is associated primarily with operational demands rather than constant severe depletion. Additionally, 64.1% of respondents agreed that shortage is a routine part of leadership responsibility (mean: 3.89±1.12). In contrast, lower agreement was observed for indicators of severe or frequent shortage. The highest- and lowest-scoring items for this domain are summarized in Table 6. 

**Table 6:** Key findings for perception of blood component shortage Scale: items were rated on a five-point Likert scale where 1 means strongly disagree, 2 means disagree, 3 means neutral, 4 means agree, and 5 means strongly agree. Percentages are based on respondents involved in blood component shortage management (n=64).

Item	Mean±SD	Agreement (%)
Need for coordination with clinical departments and leadership	4.36±0.88	84.4
Need to prioritize patients or procedures during shortages	4.23±0.91	79.7
Repeated shortages	3.28±1.21	45.3
Disruption of clinical services	3.19±1.18	50
Red-zone inventory levels	3.11±1.15	40.6

Practices during shortage

Respondents reported high engagement in multiple operational strategies. The most frequently reported practices and the least-endorsed practices are summarized in Table 7. 

**Table 7:** Reported practices during shortage situations Scale: items were rated on a five-point Likert scale where 1 means strongly disagree, 2 means disagree, 3 means neutral, 4 means agree, and 5 means strongly agree. Percentages are based on respondents involved in blood component shortage management (n=64).

Practice	Mean±SD	Agreement (%)
Donor recruitment intensification	4.58±0.65	95.3
Close monitoring of blood inventory	4.50±0.72	92.2
Recall of repeat donors	4.41±0.78	87.5
Stricter review of transfusion requests	4.22±0.89	79.7
Obtaining blood from other hospitals	4.03±0.95	71.9
Use of institutional policies in decision-making	3.31±1.10	59.4
Deferral of elective procedures	3.25±1.15	37.5

Barriers and constraints

Respondents identified multiple barriers affecting shortage management. The most strongly perceived barriers are summarized in Table 8. 

**Table 8:** Perceived barriers affecting shortage management Scale: items were rated on a five-point Likert scale where 1 means strongly disagree, 2 means disagree, 3 means neutral, 4 means agree, and 5 means strongly agree. Percentages are based on respondents involved in blood component shortage management (n=64).

Barrier	Mean±SD	Agreement (%)
Limited donor availability	3.94±1.01	67.2
High workload	3.78±1.05	67.2
Insufficient staffing	3.75±1.07	64.1
Limited decision-making authority	3.72±1.10	64.1
High transfusion demand	3.70±1.08	67.2
Limited availability of collection supplies	3.52±1.12	59.4
Limited availability of testing reagents	3.45±1.14	57.8

Improvement strategies

Respondents demonstrated strong agreement regarding potential strategies to improve blood availability. The highest-rated improvement strategies are summarized in Table 9. 

**Table 9:** Improvement strategies to enhance blood availability Scale: items were rated on a five-point Likert scale where 1 means strongly disagree, 2 means disagree, 3 means neutral, 4 means agree, and 5 means strongly agree. Percentages are based on respondents involved in blood component shortage management (n=64).

Strategy	Mean±SD	Agreement (%)
Increasing public awareness of blood donation	4.59±0.63	95.3
Implementation of patient blood management	4.53±0.69	89.1
Establishment of a national blood service system	4.53±0.72	87.5
Increasing staffing in transfusion services	4.11±0.95	75

Subgroup analysis

Subgroup analysis revealed meaningful differences across respondent characteristics.

Respondents with postgraduate qualifications demonstrated significantly higher shortage perception scores than those with lower academic qualifications (3.82 vs. 3.33; p=0.021). They also showed significantly greater recognition of repeated shortages (p=0.022), disruption of clinical services (p=0.020), high workload (p=0.008), and limited decision-making authority (p=0.012). Respondents with primary leadership responsibility reported higher barrier scores (3.87 vs. 3.39) and greater recognition of donor limitation (p=0.015) and workload (p=0.026). Older and more experienced respondents demonstrated consistent directional trends toward greater engagement in practices and stronger awareness of system constraints.

Summary of findings

Overall, the results demonstrate that blood shortage is perceived as a significant operational challenge, that respondents actively implement multiple shortage management strategies, that system-level barriers persist, including workforce, demand, and governance constraints, and that there is strong consensus on integrated improvement strategies, particularly PBM and system-level coordination.

## Discussion

The present study provides a comprehensive analysis of the factors influencing the availability of blood components from the perspective of transfusion service personnel directly involved in shortage management. The findings demonstrate that blood component shortage is not perceived as a simple issue of insufficient donor supply, but rather as a complex, multi-dimensional operational and system-level challenge, shaped by the interaction of supply limitations, clinical demand, workforce capacity, and institutional governance [8,10,11].

One of the most important findings of this study is that respondents primarily experience blood shortage through its operational consequences, particularly the need for continuous coordination with clinical teams and the prioritization of patients and procedures. These findings suggest that the burden of shortage extends beyond inventory levels and is deeply embedded in clinical decision-making processes. This aligns with existing literature, which emphasizes that modern transfusion services increasingly operate under conditions of constrained resources requiring active stewardship and prioritization, rather than simply maintaining adequate stock levels [10,11].

Interestingly, indicators of severe shortage, such as repeated depletion or "red-zone" inventory states, were reported with moderate agreement, suggesting that while catastrophic shortages may not be constant, the pressure of potential shortage is persistent. This supports the conceptualization of blood shortage as a chronic operational condition, rather than an episodic crisis. Similar patterns have been described in previous studies, where healthcare systems function under ongoing strain due to fluctuating supply and demand, necessitating continuous adjustment rather than intermittent emergency response [10,11].

The practices reported by respondents further reinforce this interpretation. The study demonstrates a high level of engagement in multi-layered operational strategies, including donor recruitment, donor recall, inventory monitoring, stricter review of transfusion requests, and inter-hospital coordination. This combination of strategies indicates that transfusion services adopt both supply-side and demand-side approaches in managing shortages. Such findings are consistent with the growing recognition in transfusion medicine that effective blood management requires a balance between increasing supply and optimizing utilization [8,10-15].

However, an important observation is the relatively lower reliance on policy-based decision-making compared to operational actions. This suggests that real-time management of shortages may depend more on clinical judgment and situational decision-making than on strictly defined institutional protocols. This finding highlights a potential gap between policy frameworks and practical implementation, which has also been reported in previous studies where guidelines are not always sufficiently adaptable to the dynamic nature of clinical environments [10,11]. Addressing this gap may require the development of more flexible, context-sensitive policies that better support frontline decision-making.

The barriers identified in this study provide critical insight into why shortages remain difficult to manage despite active operational efforts. While limited donor availability was identified as a major constraint, it was closely followed by high workload, insufficient staffing, high transfusion demand, and limited decision-making authority. This pattern clearly indicates that blood shortage is not driven by a single factor, but rather by a convergence of system-level pressures [8,10,11].

The identification of workforce-related barriers is particularly important. Shortage management requires increased coordination, monitoring, and communication, all of which place additional demands on already stretched staff. This finding suggests that even in settings where donor supply may be improved, the effectiveness of shortage management will remain limited without adequate human resources and operational capacity. Similar conclusions have been drawn in healthcare systems research, where workforce constraints are recognized as a major determinant of service performance and resilience [10,11]. 

Another key finding is the role of transfusion demand as a significant barrier. High clinical demand, particularly in tertiary care settings, may exceed available supply even under otherwise stable conditions. This underscores the importance of PBM as a central strategy for addressing shortages [12-14]. The strong support for PBM observed in this study reflects a growing awareness that improving blood availability requires not only increasing supply but also ensuring appropriate and efficient utilization of blood components [8,10-16].

Perhaps one of the most conceptually significant findings is the identification of limited decision-making authority as a barrier. This highlights the importance of organizational and governance structures in shaping the effectiveness of shortage management. Respondents may be aware of necessary interventions but lack the authority to enforce them across departments or within institutional hierarchies. This finding elevates the discussion from operational challenges to issues of leadership, policy enforcement, and institutional empowerment, which are often underrepresented in discussions of blood shortage [10,11].

The improvement strategies identified by respondents further support the interpretation of shortage as a system-level issue. While increasing public awareness of blood donation was strongly endorsed, equally high support was observed for the implementation of PBM programs and the establishment of national blood service systems. This demonstrates that respondents recognize the need for integrated solutions that address both supply and demand, as well as coordination across institutions [8,10-12].

The strong support for national blood service systems is particularly relevant in the context of predominantly hospital-based supply models observed in this study. Decentralized systems may lead to variability in resource availability and limited ability to redistribute blood components efficiently. Centralized or coordinated systems, on the other hand, may improve equity, efficiency, and responsiveness, particularly during periods of increased demand or localized shortage [10,11,17].

Subgroup analysis adds further depth to these findings by demonstrating that perception of shortage and recognition of barriers are influenced by experience, qualification, and leadership role. Respondents with postgraduate qualifications and those in leadership positions demonstrated greater awareness of shortage complexity and systemic constraints. This suggests that deeper involvement in decision-making processes enhances the understanding of the multifactorial nature of blood shortage.

At the same time, the consistent directional patterns observed with increasing age and experience indicate that exposure to shortage situations contributes to operational maturity and awareness, even when not always reflected as statistically significant differences. This highlights the importance of considering both quantitative and contextual interpretation in SPSS analysis.

Taken together, the findings of this study support a conceptual model in which blood component shortage is understood as a continuous, multi-dimensional healthcare management challenge, rather than a single-resource deficiency. Effective management requires coordinated action across multiple domains, including donor recruitment and retention, demand optimization through PBM, workforce strengthening, institutional policy development, leadership empowerment, and system-level coordination [8,10-12].

From a practical perspective, these findings have important implications for transfusion service leaders and healthcare policymakers. Efforts to improve blood component availability should move beyond isolated interventions and instead adopt a comprehensive systems approach, integrating operational, clinical, and governance strategies [8,10-12,16-19].

From an academic perspective, this study contributes to the growing body of literature that emphasizes the need to view blood shortage within the broader context of health system performance and resilience [10,11]. By focusing on the perspectives of transfusion service leaders, the study provides valuable insight into the real-world challenges and decision-making processes that shape blood availability.

This study has several limitations that should be considered when interpreting the findings. First, the study used a cross-sectional, questionnaire-based design, which captures perceptions and reported practices at a single point in time and does not allow the assessment of causality or changes in shortage management over time. Second, participants were recruited using a convenience sample from tertiary healthcare institutions in Riyadh, which may limit the generalizability of the findings to other regions of Saudi Arabia, other hospital settings, or non-tertiary transfusion services. Third, the findings are based on self-reported responses from transfusion service personnel and are therefore subject to response bias and variation in individual interpretation of shortage experiences and institutional practices. Fourth, although the principal analytical sample included respondents who were directly involved in shortage management, the sample size remains modest and was primarily suitable for exploratory and subgroup analyses rather than broad national inference. Finally, the study focused on the perceptions of transfusion service personnel and did not directly link these views to objective institutional data such as inventory records, wastage rates, or real-time blood utilization metrics. These findings should therefore be interpreted as reflecting respondents' perceptions and experiences rather than direct objective measurements of blood inventory levels, transfusion demand, staffing adequacy, or supply-chain performance. Despite these limitations, the study provides valuable experience-based insight into the multi-dimensional nature of blood component shortage management in high-demand tertiary healthcare settings and identifies practical areas for future research, policy development, and system improvement.

In conclusion, the findings demonstrate that addressing blood component shortages requires a shift from reactive, supply-focused approaches toward proactive, integrated, and system-oriented strategies. Such an approach has the potential to enhance both the efficiency and sustainability of blood component availability in modern healthcare systems [8,10-12].

## Conclusions

This study demonstrates that blood component shortage in tertiary healthcare institutions in Riyadh is not perceived as a narrow donor-supply problem, but as a broader system-level challenge shaped by the interaction of donor availability, clinical demand, workforce capacity, operational pressure, and institutional authority. Respondents reported active engagement in shortage management practices, particularly donor recruitment, close inventory monitoring, donor recall, and inter-hospital coordination, indicating that transfusion services are already responding proactively rather than waiting for critical depletion. At the same time, the persistence of barriers such as limited donor availability, high workload, insufficient staffing, high transfusion demand, and restricted decision-making authority shows that operational effort alone is insufficient when structural constraints remain unresolved. The strongest consensus was observed in the domain of improvement strategies, where respondents strongly endorsed increasing public awareness of blood donation, implementation of patient blood management, strengthening of transfusion service staffing, and development of national or more coordinated blood service models. Together, these findings support a shift from reactive shortage response toward integrated, system-oriented strategies that combine donor engagement, demand optimization, workforce strengthening, empowered leadership, and coordinated service planning to improve the resilience, efficiency, and sustainability of blood component availability.
